# Atomically-defined on-surface synthesis of multilayer metal-organic frameworks

**DOI:** 10.1038/s42004-025-01742-5

**Published:** 2025-11-18

**Authors:** Zdeněk Jakub, Jakub Planer, Dominik Hrůza, Zdeněk Endstrasser, Pavel Procházka, Jan Čechal

**Affiliations:** 1 CEITEC – Central European Institute of Technology, Brno University of Technology, Brno, Czechia; 2 Institute of Physical Engineering, Faculty of Mechanical Engineering, Brno University of Technology, Brno, Czechia

**Keywords:** Metal-organic frameworks, Surfaces, interfaces and thin films

## Abstract

On-surface synthesis allows the design and study of new materials with unrivaled resolution, but it is traditionally limited to materials of monolayer thickness. Here, we present on-surface-prepared multilayer Fe-TCNQ metal-organic framework (MOF), demonstrating that this limitation can be overcome. The multilayer Fe-TCNQ features both in-plane and out-of-plane chemical bonding, as proven by high-resolution Scanning Tunneling Microscopy and Density Functional Theory computations. The coordination geometry and electronic structure of the embedded Fe cations are affected by the interlayer chemical interaction, making the properties of the multilayer MOF distinct from the monolayer case. Furthermore, we demonstrate how the support defines the growth mode of the multilayer MOF: we observe Volmer-Weber growth mode on a graphene/Ir(111) support, but Stranski-Krastanov mode on Au(111). Our work presents new opportunities for on-surface approaches to 3D MOF synthesis and paves the way for atomic-scale characterization of these important materials.

## Abstract (web summary)

On-surface synthesis is traditionally limited to 2D materials, while the extension to 3D structures has been largely elusive. Here, the authors overcome this by synthesizing a multilayer Fe-TCNQ metal–organic framework, providing atomic-scale insights and revealing distinct growth modes on different supports, opening new avenues towards 3D materials design.

## Introduction

On-surface synthesis combined with surface science analysis presents an exceptionally successful approach to elucidating the fundamental aspects of material design^1,2^. The solvent-free deposition in ultrahigh vacuum guarantees the highest purity and perfect definition, while the surface science methodology allows probing of the physical and electronic structure of the synthesized material with unparalleled resolution. This synergy has allowed many recent breakthroughs in materials design, such as the advent of atomically-defined graphene-based nanomaterials^3–5^, demonstration of quasi-crystalline 2D materials^6,7^, development of 2D materials with designed topological states^8–10^ or resolving the finest details in charge distribution within molecular building blocks^11,12^.

However, one major limitation of the surface science approach to materials design has traditionally been its restriction to materials of monolayer thickness. This can be illustrated on the case of 2D metal-organic frameworks (MOFs), a highly promising class of materials residing at the forefront of attention of both surface scientists and materials/application chemists^13–19^. While the surface science community provides a deep understanding of the structure-function relationships of monolayer 2D MOFs^2,17,20–25^, these properties are often affected by the presence of the underlying support, whose presence is necessary for the surface science methods^20,26^. In contrast, the 2D MOF materials studied in applied materials chemistry are usually “(ultra)thin”, but not always “monolayer thin”; thus, their properties are often affected by the covalent or non-covalent interactions between the individual layers. To get the fundamental atomic-scale insights into these application-relevant materials, it is imperative to master the synthesis of multilayer structures using an on-surface approach.

Yet, despite intense efforts in the community, atomically-resolved studies aimed at on-surface growth of 3D MOFs (i.e., metal-organic structures with both in-plane and out-of-plane chemical bonding) are scarce, and the understanding is limited due to the complex multi-dimensional space of the 3D growth parameters and different kinetic barriers for in-plane and out-of-plane complexation. In liquid environment, the synthesis of 3D MOF structures on surfaces involves stabilization of linker molecules in an upright-standing configuration^27–29^, but this is much harder to do in ultrahigh vacuum. Moreover, the upright-standing species are difficult to unambiguously identify by scanning probe microscopy, which further complicates the elucidation of local atomic-scale details. Recent literature indicates that layer-by-layer growth or atomically-ordered metal-organic structures may be achievable utilizing specific “pillar molecules”, whose shape prevents them from lying flat on the surface^30–32^, but such out-of-plane complexation has so far been only demonstrated on self-assembled monolayers without the in-plane chemical bonding; these do not present 3D MOF structures. An alternative approach was recently demonstrated using a self-assembled layer of organic molecules acting as a template for fullerenes, which were subsequently cross-linked by thermally activated cycloaddition^33^. Small islands of covalently-linked structures were prepared this way, but it is difficult to imagine that a similar protocol could be scalable to thicker 3D structures or MOFs.

**Fig. 1:**
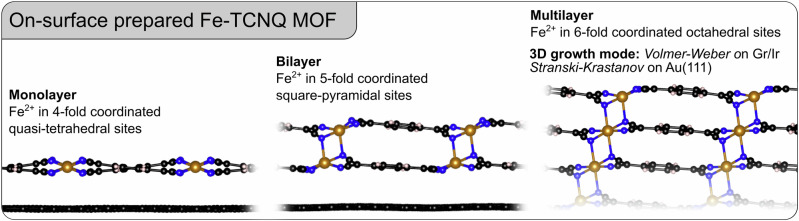
Outline of the main findings. This work shows that the Fe-TCNQ can be prepared on-surface as a monolayer 2D MOF, but also as a bi-layer and multilayer MOF, whose structure features interlayer chemical bonding. The out-of-plane bonding significantly changes the coordination geometry and electronic structure of the embedded Fe^2+^ cations, making the properties of multilayer MOF distinct from the monolayer. Such 3D MOFs can be prepared on various supports including graphene and Au(111), but the differences in the MOF/support interaction strength result in distinct 3D growth modes.

Here, we demonstrate atomically-defined 3D growth of a Fe-TCNQ MOF (TCNQ = 7,7,8,8-tetracyanoquinodimethane) on two distinct supports, graphene/Ir(111) and Au(111). As illustrated in Fig. 1, the structure of the synthesized material features both in-plane and out-of-plane chemical bonding, resulting in a 3D MOF structure synthesized by an on-surface approach in ultrahigh vacuum. The interlayer interaction changes the coordination number and electronic structure of the embedded Fe cations, making the properties of the multilayer distinct from the monolayer. The 3D MOF structure is independent of the underlying support, as the same atomic-scale structures were prepared on both graphene and gold. Different growth modes were observed on the two supports, but in both cases, the growth is scalable to thicker films, with no indication of any limitations that could prevent reaching film thicknesses well above the 3–4 layers that are shown in this work. These results pave the way towards atomic-scale understanding of not only selected 2D model systems, but also of real materials that show great promise in catalysis^34–36^ and spintronics^37,38^.

## Results and Discussion

### Synthesis of monolayer and multilayer Fe-TCNQ

**Fig. 2:**
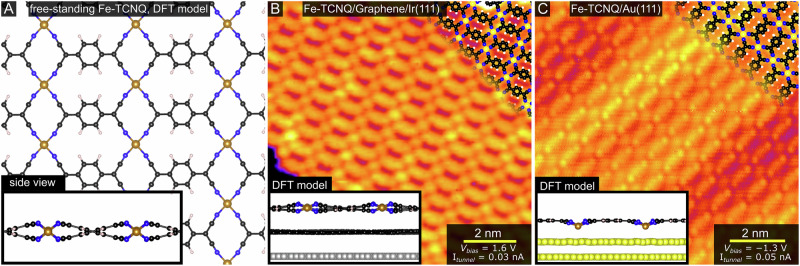
The structure of Fe-TCNQ 2D MOF in the gas phase and on graphene/Ir(111) and Au(111) supports. **A** A DFT-optimized model of free-standing Fe-TCNQ. A significant tilt of the TCNQ linkers is visible in the side view (inset), which originates from the preference of the high-spin Fe^2+^ to reside in tetrahedral coordination environment. **B** A room temperature STM image of Fe-TCNQ/graphene/Ir(111). A corresponding DFT model is shown in the inset. **C** A room temperature STM image of Fe-TCNQ/Au(111), along with a corresponding DFT model shown in the inset. A detailed analysis of the monolayer Fe-TCNQ structures is provided in ref. ^20^.

Figure 2A shows a density functional theory (DFT) model of monolayer Fe-TCNQ network in the gas phase, panels B and C show scanning tunneling microscopy (STM) images and corresponding DFT models of the same 2D MOF synthesized on graphene/Ir(111) and on Au(111). On both supports, the 2D MOF was synthesized by co-deposition of TCNQ molecules and Fe atoms at a temperature close to the TCNQ desorption (see Methods for details). Following the co-deposition step, the samples were post-annealed to 340 °C (on Au) or up to 420 °C (on graphene). On both supports, the 2D MOF forms a similar structure with fourfold coordinated Fe atoms (Fe-N_4_ sites), evidenced by the similar appearance in STM and low-energy electron diffraction (LEED)^20^. Yet, these systems are not structurally identical: our previous work indicates that on graphene/Ir(111) the system is non-planar and accommodates the Fe-N_4_ site preferentially in a quasi-tetrahedral geometry, while on Au(111) it is planarized by stronger van der Waals interaction which draws the Fe cations closer to the Au surface^20^. This difference is clearly visible in the side views of the DFT-optimized structures shown in the insets of Fig. 2B, C, and also in STM images taken at specific bias voltages. Qualitatively similar structures were also observed for other 2D MOFs with TCNQ linker molecules, although the non-planarity of the structure was only addressed in some works^39–43^.

**Fig. 3:**
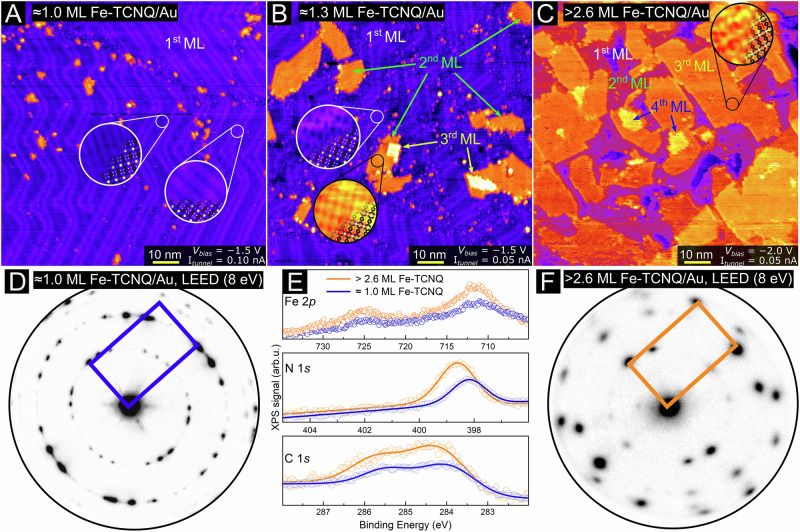
Synthesis and characterization of Fe-TCNQ multilayers on Au(111). **A** Large-scale room-temperature STM image of a monolayer Fe-TCNQ atop Au(111). The image features differently oriented rotational domains of Fe-TCNQ. Small islands of additional material are observed at the domain boundaries of the Fe-TCNQ rotational domains. **B** An STM image of multilayer Fe-TCNQ islands atop a closed Fe-TCNQ monolayer on Au(111). The periodicity measured on the islands is the same as that of the Fe-TCNQ monolayer. **C** A true multilayer structure of Fe-TCNQ on Au(111). Here, 90% of the surface is covered by a Fe-TCNQ bilayer, while 60% is covered by a tri-layer. Fe-TCNQ covers the whole surface, the small black area in the top right corner is Fe-TCNQ on a lower Au(111) terrace. **D** LEED pattern of the Fe-TCNQ monolayer shown in panel A. The Fe-TCNQ unit cell in reciprocal space is highlighted by the blue rectangle. **E** XPS spectra of the Fe-TCNQ monolayer (blue) compared to the spectra of the multilayer (orange). **F** LEED pattern of the Fe-TCNQ multilayer. The unit cell is highlighted by an orange rectangle, and is identical to the one found on the monolayer case shown in panel D.

**Fig. 4:**
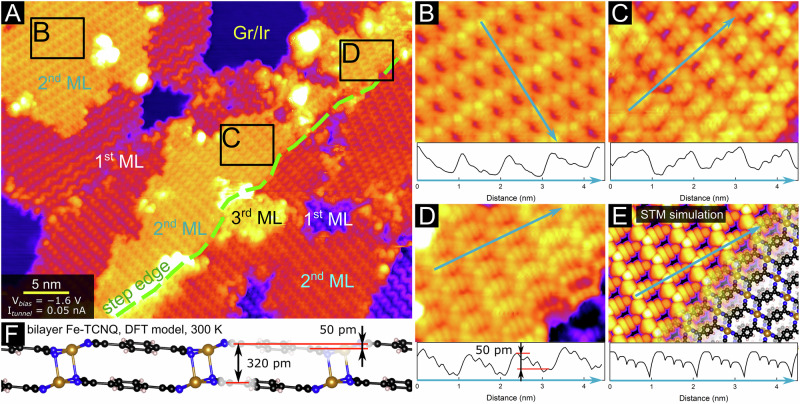
STM characterization of Fe-TCNQ multilayers on graphene/Ir(111) and comparison to DFT model. **A** An overview room-temperature STM image of the Fe-TCNQ/graphene/Ir(111) system. The image features areas of bare graphene/Ir(111) (labeled Gr/Ir) as well as areas of Fe-TCNQ monolayer (labeled 1st ML), bi-layer (2nd ML) and tri-layer (3rd ML). A step edge in the Ir(111) support is highlighted by green dashed line. **B**–**D** Details of the Fe-TCNQ bi-layer structure. All these images were cut from the same STM image acquired with the same tip. The structure of the Fe-TCNQ bi-layer has a distinct appearance, in which all the TCNQ linkers within an island show the same apparent tilt, as highlighted by the line scans shown in the bottom part of each panel. **E** An STM simulation (V_*bias*_ = −1.2 V) based on the DFT model shown in (**F**) shows excellent agreement with the experimental STM images shown in (**B**–**D**).

Figures 3 and 4 provide unambiguous evidence that the on-surface synthesis protocol for well-ordered Fe-TCNQ growth is not limited to sub-monolayer coverages but can be scaled up to thicker layers. First, Fig. 3A shows an STM image of 1 monolayer (ML) of Fe-TCNQ on Au(111). Here, the Fe-TCNQ monolayer is rather perfect, and a small amount (<0.05 ML) of Fe-TCNQ material is residing on top. The periodicity of the Fe-TCNQ monolayer is visible in zoomed-in insets, and is also clearly identified in the LEED pattern shown in Fig. 3D. The LEED data further indicate that the Fe-TCNQ monolayer is present in nine dominant orientations on the Au(111) support, as analyzed in detail in a previous study^20^. The presence of Fe-TCNQ is also detected by XPS (blue spectrum in Fig. 3E) where the signal in the Fe 2p, N 1s and C 1s regions is clearly observed. Fitted XPS spectra are provided in Supplementary Note 1; the peak positions and shapes are fully consistent with similar systems reported in literature^39,44,45^. Specifically, the Fe 2p region features a complex multiplet structure with a Fe 2p_3/2_ maximum around 711.5 eV, while the N 1s and C 1s regions can be fitted by a single N 1s and four C 1s components, as expected for TCNQ molecules^46,47^. Importantly, the absence of a characteristic shake-up satellite in the N 1s and C 1s regions rules out presence of neutral TCNQ^44,45,47,48^, providing further evidence that the TCNQ linkers are complexed with Fe cations. The LEED and STM data further indicate significant interaction between the Fe-TCNQ and Au support, which is manifested by the varying periodicity of the herringbone reconstruction of the Au(111)^20^.

Figure 3B shows an STM image of a Fe-TCNQ monolayer on Au(111) with well-resolved islands of the 2^nd^ and 3^rd^ Fe-TCNQ layers on top. The sample was synthesized by the same protocol as the one in Fig. 3A, only the co-deposition step was prolonged. The periodicity of the 2^nd^ and 3^rd^ Fe-TCNQ layers appears very similar to the periodicity of the monolayer, as shown in the zoomed-in insets. Interestingly, the herringbone reconstruction of the Au(111) support is still visible under the bi-layer Fe-TCNQ islands (see Supplementary Note 2).

Finally, Fig. 3C shows an STM image of a true multilayer Fe-TCNQ structure. Here, more than 90% of the Au(111) surface is covered by at least a Fe-TCNQ bi-layer, while more than 60% of the measured area is covered by a Fe-TCNQ tri-layer. A few small islands of a quad-layer are also observed, indicating that the growth does not terminate here, and can be scaled up further to thicker layers. Importantly, no large Fe clusters are observed in STM, and the XPS signal also does not detect any significant signal originating from metallic Fe (orange curves in Fig. 3E).

The XPS spectra of monolayer and multilayer Fe-TCNQ (blue and orange spectra in Fig. 3E) look very similar with the only obvious differences being the lower intensity of the monolayer and a ≈0.3 eV shift in peak positions (see Supplementary Note 1 for details). This shift is most likely of electrostatic origin, caused by different vertical charge distribution at the interface^49^. The LEED data shown in Fig. 3F indicate that the unit cell of the multilayer Fe-TCNQ structure looks identical to the monolayer one, but there is a difference in the number of symmetry-equivalent domains present on the surface. Specifically, the multilayer Fe-TCNQ/Au does not feature the domains aligned with the high-symmetry directions of Au(111), which were previously observed on the monolayer samples (additional data shown in Supplementary Note 3). The exact reason for the reduction in the domain count is currently unknown, but it seems likely that it may be driven by minimization of strain energy at the MOF-support interface. In a multilayer Fe-TCNQ/Au structure, the MOF-support interaction is expected to be weakened by the so-called “surface trans effect”^50,51^. Our computational results support such a scenario (see Supplementary Note 10), and the weaker MOF-support interaction may allow easier rearrangement to a structure with lower strain at the Fe-TCNQ/Au(111) interface.

### Atomic-scale structure of multilayer Fe-TCNQ

The information about the presence of interlayer chemical bonding within the MOF structure can be obtained by STM imaging of the MOF on graphene/Ir(111), where the achievable STM resolution is higher due to decoupling from the metal support. On the graphene support, the Fe-TCNQ is observed in 15 rotational domains (see Figure S6 in Supplementary Note 3), and multilayer structures are observed already prior to closing of the first monolayer, thus the image in Fig. 4A shows areas of clean graphene/Ir(111) as well as areas of Fe-TCNQ monolayer, bi-layer and tri-layer. These individual regions are labeled in Fig. 4A; the image also features a step edge of the underlying Ir(111) support (highlighted by a dashed green line). In parts of the Fe-TCNQ monolayer regions, one can clearly resolve the characteristic “zig-zag” patterns, which are present when the tilt angles of the neighboring non-planar TCNQ linkers are ordered in alternating fashion (as described in detail in ref. ^20^). In contrast, the Fe-TCNQ bi-layers and tri-layers show a distinct, more homogeneous appearance.

In detailed zoomed-in STM images acquired at a negative sample bias (Fig. 4B–D), all the TCNQ molecules look alike, with four lobes along their longer high-symmetry axis. This is consistent with the appearance of the TCNQ LUMO^52,53^, which is occupied in the Fe-TCNQ structure due to the chemical bonding and charge transfer from Fe^20,39^. Importantly, the STM line profiles shown in panels B-D all indicate a clear unidirectional tilt of the TCNQ linkers, with the four lobes of each individual TCNQ LUMO having different apparent height. The apparent height of the individual lobes is ordered in a linearly increasing or decreasing pattern, and the difference between the highest and lowest lobe of a single TCNQ is between 50–70 pm. Within a single Fe-TCNQ bilayer island, all the TCNQ molecules are tilted in the same direction, as shown in Fig. 4B–D and Fig. S7. This is consistently observed in filled-states STM images of bi-layer and tri-layer islands taken on various spots on the surface with different STM tip contrasts (details in Supplementary Note 4), providing clear experimental evidence that the multilayer structures are structurally distinct from the monolayers.

The origin of the distinct appearance of multilayer Fe-TCNQ is elucidated by DFT computations, which reveal that the unidirectional tilt observed in experiment is a direct consequence of interlayer chemical bonding. Figure 4E shows an STM simulation of the DFT-optimized structure depicted in Fig. 4F, which perfectly reproduces the experimental STM images. Moreover, the height difference between the end groups of the tilted TCNQ molecules is about 50 pm, in agreement with the experimental measurements.

**Fig. 5:**
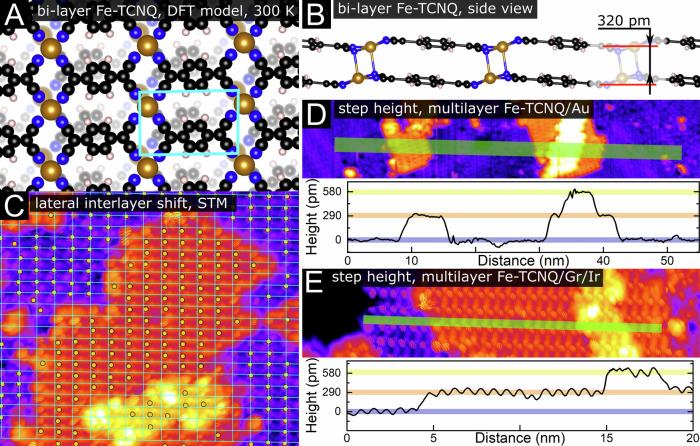
Interlayer shift and step height within Fe-TCNQ bi-layer structure. **A, B** Top and side views of the DFT-optimized model of the lowest-energy Fe-TCNQ bi-layer structure; the atom positions were averaged from a molecular dynamics simulation at 300 K. The model features a significant interlayer shift originating from the chemical bonding between the Fe cations and -CN groups in the stacked layers. This interlayer shift is also observed in STM images of Fe-TCNQ/Gr/Ir, as shown in (**C**). Here, the Fe atom positions were labeled by an automated script (yellow points), and a cyan grid was overlaid to highlight the positions of the Fe atoms in the first Fe-TCNQ layer. The Fe atom positions in the 2^nd^ and 3^rd^ layer are visibly shifted from the Fe positions in the 1^st^ layer. **D**, **E** Analysis of the step height of multilayer Fe-TCNQ structures on graphene and Au. In both cases, a step height of (290 ± 30) pm is found, close to the value found in the DFT model shown in panel **B**.

A detailed top view on the DFT model is provided in Fig. 5A. The ground state structure was identified by DFT computations, and the model was subsequently re-computed by molecular dynamics using machine-learned force fields (MD-MLFF) to represent an averaged structure measured at room temperature. The model features strong interlayer CN-Fe chemical bonding: In the 0 K ground state, the interlayer bond length is 2.19 Å, which is comparable to the 2.03–2.25 Å found within the individual Fe-TCNQ layers. The covalent nature of the interlayer bonding is further evidenced by the Crystal Orbital Hamilton Population (COHP)^54^ analysis provided in Supplementary Note 5. Alternative models of Fe-TCNQ bi-layers were tested both in gas-phase and on surfaces, and the spontaneous formation of interlayer chemical bonding was observed in most of them (see Supplementary Notes 6-9). Stable bi-layer structures without interlayer bonding were also found, but their presence in the experiment can be ruled out based on their very distinct STM appearance (see Supplementary Note 6)

Another significant structural feature of the bi-layer Fe-TCNQ model is a lateral interlayer shift caused by the preference of the Fe in the top layer to reside directly above N atoms in the underlying layer. The interlayer shift is clearly observed in both in DFT models and in STM images: In Fig. 5C, the positions of Fe atoms were labeled by yellow dots using an automated script, and a cyan grid was overlaid to highlight the Fe atom positions in the 1^st^ layer. The Fe atoms in the 2^nd^ layer are shifted with respect to the 1^st^ layer, and a shift of similar direction and magnitude is also observed between the 2^nd^ and 3^rd^ layer. Such interlayer shifts were consistently observed in high-resolution STM images, although the directions and magnitudes of these shifts slightly varied. This is consistent with our DFT computations showing that interlayer shift variations in the sub-Ångström range affect the bi-layer stability only marginally (details in Supplementary Note 7).

The step height measurements are consistent between the STM images and DFT models. A height difference of 320 pm is found in the room-temperature averaged MD model between the equivalent atoms in the neighboring layers (Fig. 4F; in the 0 K ground state it is 340 pm). As shown in Fig. 5D, E, this value is close to the step heights measured by STM on both supports, where a height difference of (290 ± 30) pm was measured between neighboring Fe-TCNQ layers.

**Fig. 6:**
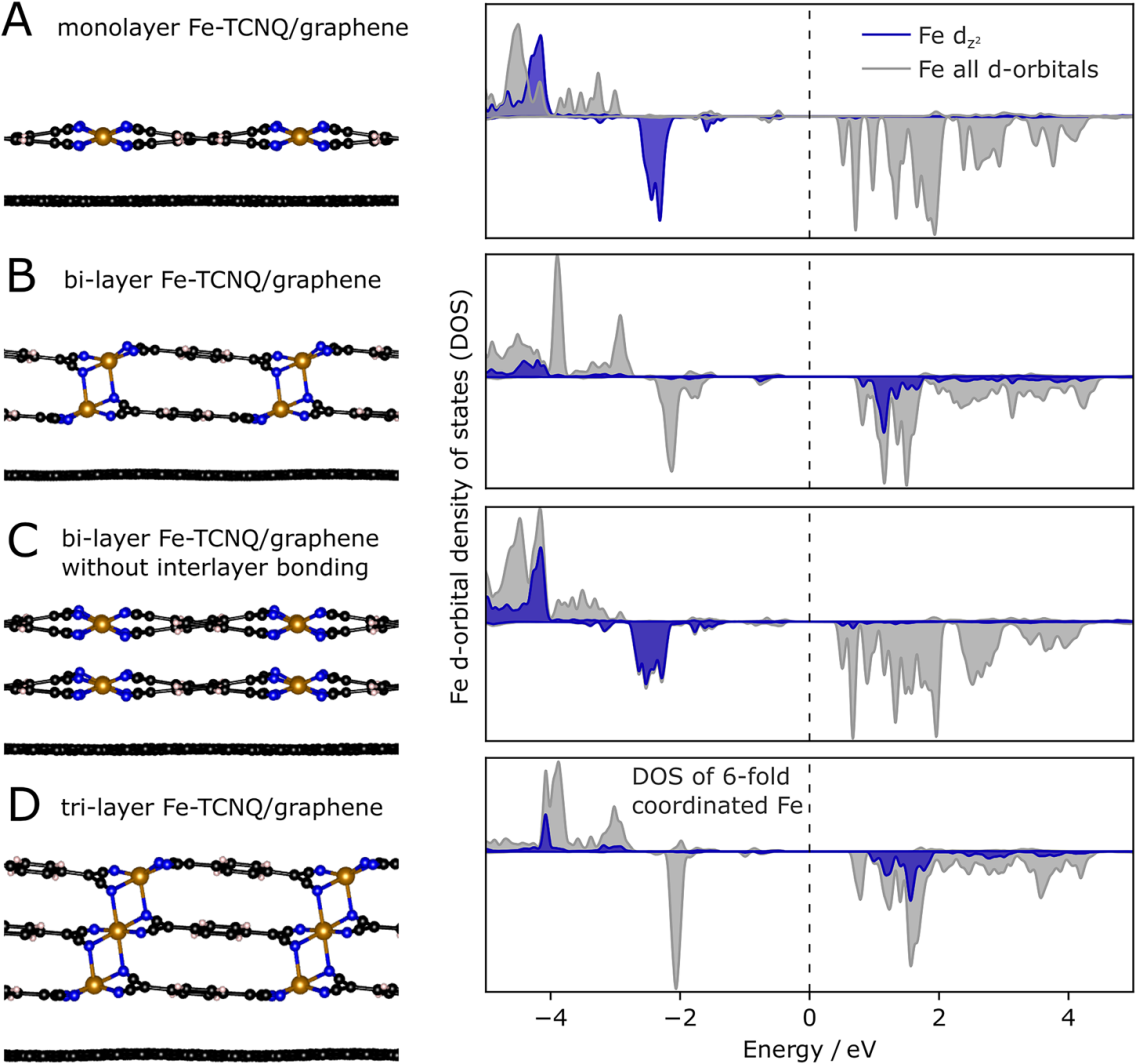
Differences in the electronic structure of the monolayer and multilayer Fe-TCNQ models. In all cases the Fe atoms remain in high-spin d^6^ state, but significant differences are observed in the individual occupancies of the d-orbitals. Specifically, the ${{{\rm{d}}}_{{{\rm{z}}}^2}}$ orbital is fully occupied in the graphene-supported monolayer (**A**), but only half-occupied in the multilayer case (**B**). This change in the ${{{\rm{d}}}_{{{\rm{z}}}^2}}$ occupancy is caused by the interlayer chemical bonding; the hypothetical structure without such bond has the same properties as the monolayer (**C**). In Fe-TCNQ structures thicker than a bi-layer, the Fe cations are 6-fold coordinated (**D**), and their electronic structure is very similar to the 5-fold Fe.

Our experiments focused mostly on the bi-layer and tri-layer Fe-TCNQ MOFs, but it can be safely assumed that thicker layers will feature the same structural motifs as these thin models. Our experimental data show that the tri-layers have identical STM appearance to bi-layers, both in terms of registry matching with respect to lower-lying layers, and in terms of the characteristic unidirectional tilt of the TCNQ linkers (see Fig. 5 and Supplementary Note 4). No evidence of alternative structures was identified in the experimental dataset. This is consistent with our DFT results of multilayer Fe-TCNQ models (Fig. 6) which indicate that the interlayer bonding motif remains qualitatively the same in all these models, as the Fe cation coordination evolves from 4-fold quasi-tetrahedral in the monolayer through 5-fold distorted square-pyramidal in the bi-layer to 6-fold octahedral in the tri-layer and thicker structures. Interestingly, this change in local coordination does not affect the charge or spin state of the Fe cations, which remain in a high-spin d^6^ state, same as in the monolayer Fe-TCNQ^20,55^. It does, however, change the occupancy of the individual Fe orbitals, as depicted in the projected density of states (DOS) plots of the Fe d-orbitals in Fig. 6. A monolayer Fe-TCNQ on graphene (panel A) has a fully occupied Fe ${{{\rm{d}}}_{{{\rm{z}}}^2}}$ orbital, while the experimentally observed Fe-TCNQ bilayer structure (panel B) features a dominant unoccupied ${{{\rm{d}}}_{{{\rm{z}}}^2}}$ state around 1.5 eV above the Fermi level. This different d-orbital occupancy is primarily caused by the interlayer chemical bond, as evidenced by the comparison to a DOS plot of a hypothetical bi-layer structure without the interlayer bonding (panel C), which shows the same properties as the monolayer. Upon extension to thicker 3D structures, the electronic structures of the 5-fold and 6-fold coordinated Fe are very similar, both with a half-filled ${{{\rm{d}}}_{{{\rm{z}}}^2}}$ orbital (panels B, D).

### The effect of the support

Finally, we address the differences in the multilayer Fe-TCNQ systems on the two supports. We have shown that the protocol to achieve multilayer growth is at least partially support-independent, as very similar structures have been observed on Au(111) and graphene/Ir(111). The bi-layer Fe-TCNQ structures on Au(111) and graphene may slightly differ due to the higher reactivity of the Au(111) support (which draws the Fe atoms closer to the support^20^), but it is reasonable to assume that any structural differences will be localized at the MOF-support interface. Indeed, our DFT computations indicate that the lower-lying Fe atoms do not preclude formation of interlayer chemical bonding (see Supplementary Note 10); thicker MOF layers are thus expected to be unaffected by small variations of the MOF-support interface structure. However, the MOF-support interaction plays a crucial role in deciding the growth mode: On Au(111), we observe a Stranski-Krastanov (layer-plus-island) growth mode, where the 2^nd^ layer nucleates only after closing of the first monolayer, but then the islands of subsequent layers (*n* + 1) grow already at an incomplete *n*^th^ layer coverage. In contrast, on graphene/Ir(111) we observe a Volmer-Weber (island) growth mode in which the multilayers appear well before closing of the first monolayer. These observations are in line with the different relative strengths of the MOF-support and MOF-MOF interaction: The interaction of the Au(111) with both the MOF and non-coordinated TCNQ linkers is significantly stronger compared to graphene, while the binding of TCNQ adsorbed on the Fe-TCNQ/Au is weaker than on Fe-TCNQ/graphene^20,56^. This results in the first monolayer growth being preferred over the multilayer growth on the gold support. In contrast, on graphene, the MOF-support interaction is weaker, while the TCNQ adsorption atop Fe-TCNQ/graphene is stronger^20,56^, resulting in a scenario in which the 2^nd^ layer islands nucleate prior to closing of the 1^st^ layer. Nevertheless, despite these differences, the growth seems easily scalable to thicker layers on both supports, and no evidence of any alternative multilayer structures or substantial Fe clustering was observed.

## Discussion

We believe that the facile growth of atomically-defined Fe-TCNQ 3D MOF structures is primarily allowed by three aspects: (1) the fact that the out-of-plane coordination is established between co-planar layers, which bypasses the need to stabilize linkers in upright-standing configuration, (2) the fact that the linker of subsequent layers preferentially adsorb on well-defined sites on the underlying layers, as studied in detail in our previous works^20,56^, and (3) the structural flexibility of the metal center that can adopt various coordination geometries (from tetrahedral, through square pyramidal to octahedral), allowing formation of interlayer chemical bonds without breaking the in-plane coordination. Thus, we expect that similarly well-defined on-surface 3D growth will be achievable for many similar MOF systems, but it is crucial to select the right linkers and metal centers. Large linker molecules may lack a clear preference for a single adsorption geometry on an underlying MOF layer, making the ordered multilayer growth difficult. The choice of the metal center is just as important, as it can be expected that metal centers with a strong preference for square-planar coordination will be less likely to participate in interlayer chemical bonding within a multilayer MOF structure. These points illustrate the importance of detailed case studies, from which the necessary fundamental insights of the individual MOFs, linkers molecules and metal centers can be gained.

It is very likely that the out-of-plane metal-NC coordination affects the material properties that are crucial for potential applications, for example, in catalysis^34–36^ or spintronics^37,38^. Specifically, the change in coordination geometry of the metal centers and their varying d-orbital occupation will likely affect both the reactivity of the metal atoms^57–59^ and the strength of the magnetic coupling^19,40,60^. Our work sets the scene for unraveling and quantifying such effects at the atomic scale.

## Conclusions

We have demonstrated that the growth of 3D Fe-TCNQ metal-organic framework is possible using an on-surface approach, and that these multilayer structures can be readily characterized with the unparalleled resolution of the surface science toolbox. Our data indicate that the on-surface self-assembly of multilayer structures is feasible on various substrates, including the prototypical Au(111) as well as a technologically more relevant graphene. On both tested supports, the synthesis protocol is easily scalable to thicker layers, with no indications of any potential hurdles that could prevent thick-layer growth. The multilayer Fe-TCNQ is chemically bonded both in-plane and out-of-plane, and this chemical interaction changes the structural and electronic properties of the multilayer MOF with respect to the monolayer 2D MOF. These systems bridge the gap between surface science models and real systems applied in materials chemistry and pave the way towards atomic-scale understanding of applied materials.

## Methods

Experiments were conducted in several ultrahigh vacuum chambers connected by a central transfer line, separated by gate valves. The base pressure of all the chambers is below 5 × 10^−10 ^mbar. The Au(111) surface sample was prepared by repeated cycles of sputtering (1.5 kV Ar^+^, 10 min) and annealing (540 °C, 2–10 min). The graphene/Ir(111) samples were prepared by sputtering (1.5 kV Ar^+^, 10 min) and annealing (up to 1250 °C, 10 min). The first annealing cycle was carried out in a partial pressure of O_2_ and at lower temperature (1 × 10^−6 ^mbar, 1100 °C) to remove any graphene residues from the previous experiments. Graphene was grown by a protocol combining temperature-programmed growth and chemical vapor deposition^61,62^, which consistently leads to a full monolayer of well-defined graphene/Ir(111). Specifically, a saturation amount of ethylene (C_2_H_4_) was adsorbed on the bare Ir(111) surface at room temperature. After closing of the ethylene leak valve, the sample was heated to 1250 °C in ultrahigh vacuum, and ethylene was re-introduced into the chamber with the sample at this temperature (5 × 10^−7^mbar, 3 min).

TCNQ molecules were evaporated from an MBE Komponenten OEZ cell heated to 110 °C. Fe was evaporated from an MBE Komponenten HTEZ cell, the evaporation rate was checked by a water-cooled quartz crystal microbalance. The Fe-TCNQ was prepared on both surfaces by deposition of TCNQ at a sample heated to a temperature close to the TCNQ desorption (80 °C for graphene/Ir(111), 140 °C for Au(111)) and then co-deposition of TCNQ and Fe at the same temperature. The final step of the synthesis was post-annealing to 340 °C (on Au) or up to 420 °C (on graphene) for 10 min.

STM data were acquired at room temperature using a SPECS Aarhus 150 microscope, equipped with a Kolibri sensor with a tungsten tip. Where possible, the data were corrected for piezo creep and thermal drift using the method described in ref. ^63^. LEED data were acquired in a SPECS FE-LEEM P90 LEEM/PEEM instrument. XPS analysis was performed on a SPECS system equipped with a non-monochromatized Mg X-Ray source (SPECS XR50) and SPECS Phoibos 150 spectrometer. The experimental data were acquired in separate experimental runs over the timeframe of 4 years. The observation of multilayer Fe-TCNQ structures is fully reproducible; multilayers were identified in hundreds of STM images acquired after different sample preparations. Slight variations in preparation protocol (deposition rate of Fe atoms and TCNQ linkers, substrate temperature during co-deposition and post-annealing) lead to variations in the size and shape of the Fe-TCNQ islands (both monolayer and multilayer), but no significant differences in the atomic-scale structures were identified.

Spin-polarized density functional theory (DFT) calculations were carried out using the Vienna ab initio Simulation Package (VASP)^64^. In line with our previous works^20,56^, we used the nonlocal van der Waals corrected optPBE-vdW functional^65^ for the description of exchange correlation energy, and included the Hubbard-like coulomb repulsion correction *U*-*J* = 4 eV in Dudarev’s formulation^66^ for an appropriate description of Fe 3 d orbitals. The Brillouin zone was sampled using a Γ-centered Monkhorst–Pack grid^67^ with a spacing of ~0.2 Å^–1^.

Structural models for the Fe-TCNQ/Gr interfaces are consistent with ref. ^20^. To assess the impact of strain imposed by commensurating the substrate and Fe-TCNQ structures, we constructed five distinct interface models, described in Supplementary Note 9, which confirm that strain does not qualitatively change the presented results. Structural optimizations were performed until all residual forces acting on atoms were below 0.02 eV/Å. All calculations account for dipole corrections to both energy and forces.

MD simulations of the free-standing Fe-TCNQ bilayer were conducted using machine-learned force fields (MLFF) with the Gaussian potential approximation, as implemented in VASP 6.4.2. The MLFF model was trained from 872 local configurations acquired on-the-fly using the canonical NVT ensemble. The temperature of 300 K was controlled by a Nosé-Hoover thermostat with a Nosé mass set to 1.2. The presence of the graphene support was simulated by applying a harmonic potential, which acts on the z-positions of carbon atoms in the first Fe-TCNQ monolayer that comprises central rings of the TCNQ molecule. The force constant was estimated at 0.2 eV/Å^2^ by tilting the central rings in models with and without the graphene support (see Supplementary Note 8). The equilibrium position (i.e., minimum) of the harmonic bias potential was set for each constrained carbon atom to its optimal z-position obtained from the ground-state interface calculation. Finally, MD simulations using force field predictions alone were performed with the same parameters and the temperature-averaged structure was obtained by averaging over 40000 MD steps.

Scanning tunneling microscopy (STM) simulations are based on the Tersoff-Hamann approximation^68^ in the constant current mode. Isocontour value of 5 × 10^−5^ Å^-3^ of the partial electron density is displayed.

## Supplementary information

Supplementary Information

## Data Availability

The primary datasets generated in this study are available in the Zenodo repository: 10.5281/zenodo.17358822.
