# Supplementary material for: Atomically-defined on-surface synthesis of multilayer metal-organic frameworks
Source: Commun Chem. 2025 Nov 18;8:357. doi: 10.1038/s42004-025-01742-5 (PMC12627721; doi:10.1038/s42004-025-01742-5)
Supplement: Supplementary file 1 — Supplementary Information [file 42004_2025_1742_MOESM1_ESM.pdf]

Supporting Information

Atomically-defined on-surface synthesis of multilayer metal-organic frameworks

Zdeněk Jakub[1], Jakub Planer[1], Dominik Hrůza[1], Zdeněk Endstrasser[1], Pavel Procházka[1], Jan Čechal[1,2]

[1] CEITEC – Central European Institute of Technology, Brno University of Technology, Purkyňova 123, 61200 Brno, Czech Republic

[2] Institute of Physical Engineering, Faculty of Mechanical Engineering, Brno University of Technology, Technická 2896/2, Brno 61200, Czech Republic

Table of Contents

Supplementary Note 1: Fitted XPS spectra of Fe-TCNQ/Au(111) 2

Supplementary Note 2: Visibility of the Au(111) herringbone reconstruction under Fe-TCNQ bi-layer 3

Supplementary Note 3: Additional LEED and LEEM data 3

Supplementary Note 4: STM appearance of the Fe-TCNQ multilayer with different tip termination and different sample biases 6

Supplementary Note 5: DFT analysis of the inter-layer coordination bonding 7

Supplementary Note 6: Gas-phase DFT models and STM simulations 9

Supplementary Note 7: Additional STM and DFT analysis of the lateral shifts between individual Fe-TCNQ layers 10

Supplementary Note 8: Estimation of the spring constants applied in the gas-phase MD simulations 11

Supplementary Note 9: DFT models of bi-layer Fe-TCNQ supported on graphene 12

Supplementary Note 10: DFT analysis of the bilayer Fe-TCNQ structures on Au(111) 14

Supplementary Note 11: Electronic properties of the Fe-TCNQ structures supported on graphene 16

Supplementary Note 1:

Fitted XPS spectra of Fe-TCNQ/Au(111)

Figure S1 shows fitted XPS spectra presented in Figure 3 in the main text. Panels A-C show the Fe 2p, N 1s and C 1s regions of the monolayer Fe-TCNQ/Au(111), panels D-F show the same regions of the tri-layer sample presented in Figure 3 in the main text. Shirley background and Voigt function lineshape with Lorentzian width of 0.3 eV and Gaussian width of 1.2 – 1.4 eV was used in all the fits, the individual fitting parameters are given in Table ST1. The Fe 2p region was not fitted because of its notoriously complex multiplet structure that is subject to more interpretations.[1,2]

The N 1s region features a single component and an absence of shake-up satellite, in accordance with the presence of anionic or dianonic TCNQ molecule. The C 1s region features four components, as there are four distinct C atoms within the TCNQ structure. The areas of the individual components were constrained to the ratio of 2:1:1:2, in agreement with the molecular stoichiometry and with previous XPS studies of similar systems.[3] In all the regions, a small 0.2 – 0.4 eV shift is observed between the monolayer and multilayer sample; this shift likely originates from the different work function of the monolayer and multilayer systems.

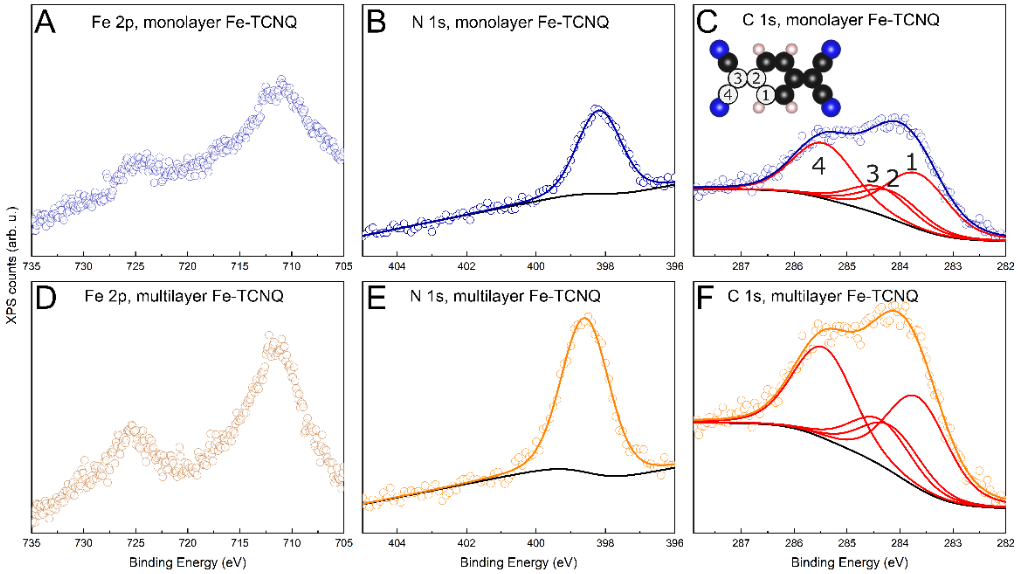

Figure S1: XPS spectra of monolayer Fe-TCNQ (A-C) and multilayer Fe-TCNQ (D-F) on an Au(111) support. Fe 2p, N 1s, and C 1s spectra were taken employing pass energy of 40, 50, and 30 eV, respectively.

Table ST1: Parameters of XPS fits of N 1s and C 1s regions shown in Figure S1.

	N	C (1)	C (2)	C (3)	C (4)
monolayer Fe-TCNQ/Au	398.2	283.7	284.2	284.4	285.5
3-layer Fe-TCNQ/Au	398.6	283.9	284.4	284.6	285.7

Supplementary Note 2:

Visibility of the Au(111) herringbone reconstruction under Fe-TCNQ bi-layer

Figure S2A shows a large-scale STM image, in which the herringbone reconstruction of the Au(111) support is clearly visible both under the monolayer Fe-TCNQ (blue-pink areas, majority of the image) and under the bi-layer Fe-TCNQ (orange areas). Panels B – E show zoomed-in insets with adjusted contrast to highlight the herringbone corrugation under the bilayer.

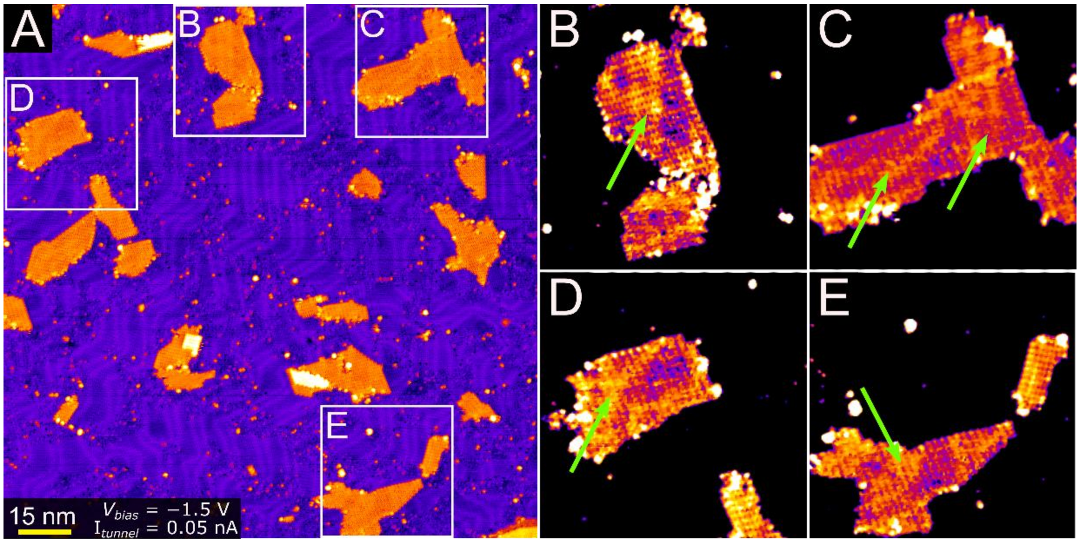

Figure S2: Herringbone reconstruction of the Au(111) surface visible under bilayer Fe-TCNQ. Panels B-E show magnified areas highlighted in panel A. The stripes and elbows of the herringbone reconstruction visible underneath the bilayer Fe-TCNQ are highlighted by green arrows.

Supplementary Note 3:

Additional LEED and LEEM data

Figure S3 shows LEED patterns of the Au(111) sample with >2.6 ML Fe-TCNQ. All patterns were acquired on the same sample, at different positions on the sample. The same Fe-TCNQ unit cell is present in all images, but with different intensity of the individual domains. The LEED patterns originate from an area of about 120 μm^2. The pattern shown in panel A features six rotational and mirror domains, patterns in B-D are missing some of these, suggesting that the areas covered by the individual domain orientations are rather large and spatially separated (as further confirmed by LEEM data shown in Figure S4).

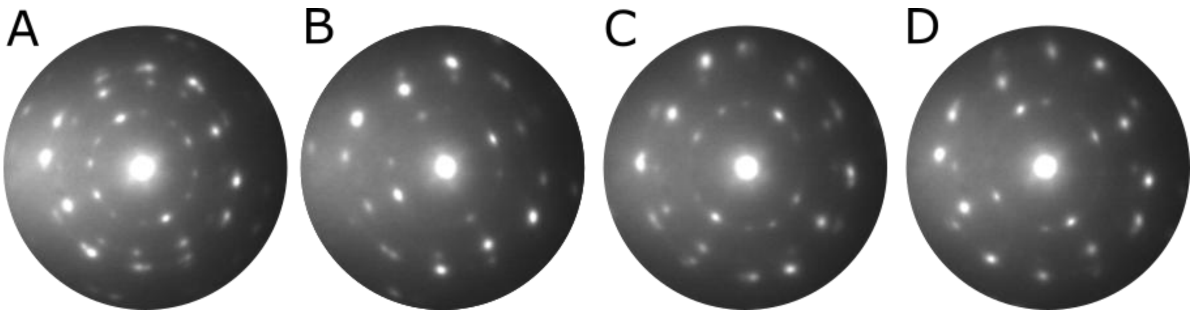

Figure S3: Additional LEED patterns of >2.6 ML Fe-TCNQ/Au(111) acquired on different places on the same sample. The primary electron energy was 17 eV in panel A,B and 16 eV in panels C,D. The same Fe-TCNQ unit cell is observed in all panels.

Figure S4 shows bright and dark field LEEM images of the Au(111) sample with ≈ 1.6 ML Fe-TCNQ. Panel A shows the bright field, panel B shows the corresponding diffraction pattern. Panels C-F then show the dark field images originating from the diffraction spots highlighted in panel B. These dark field images indicate that the areas preferentially covered by Fe-TCNQ in one specific orientation are tens of μm^2 large.

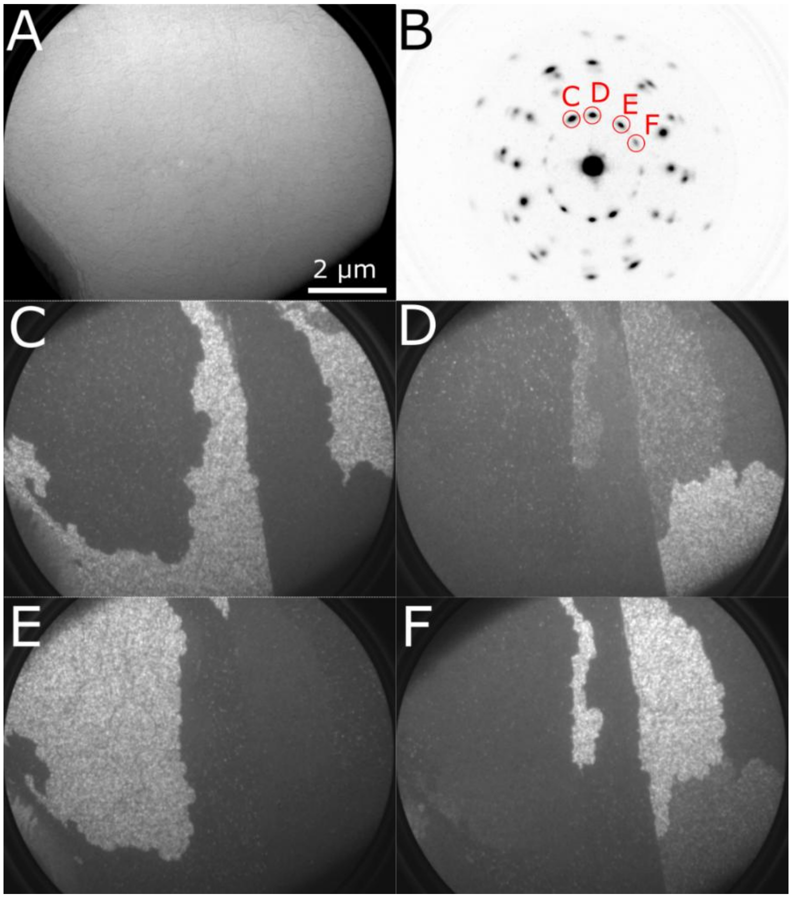

Figure S4: Bright field (A) and dark field (C-F) images of the Au(111) sample with 1.6 ML Fe-TCNQ. The diffraction spots from which the dark field images originate are highlighted in the diffraction pattern shown in panel (B).

Figure S5 shows bright field LEEM-I(V) fingerprints of the Fe-TCNQ layers of different thicknesses on Au(111). The I(V) curves clearly show significant differences with increasing layer thickness, suggesting the possibility of estimating the number of layers from LEEM measurements.

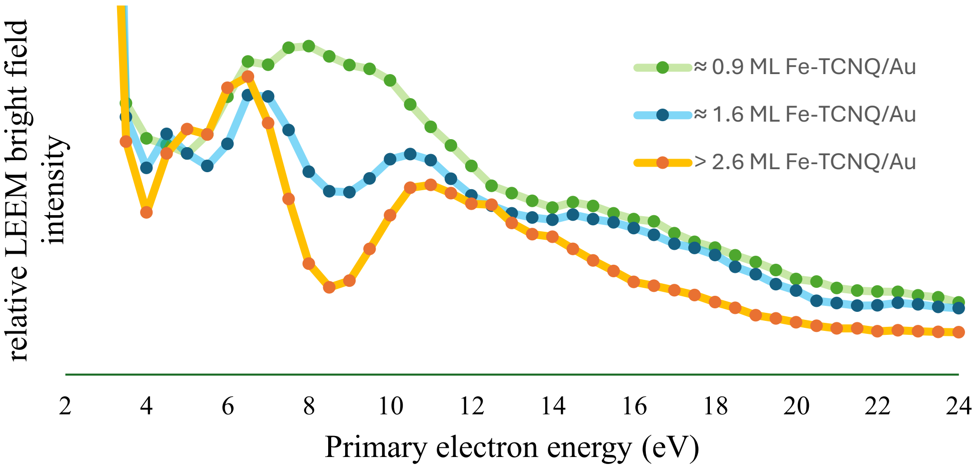

Figure S5: LEEM-I(V) fingerprints of Fe-TCNQ layers with different thicknesses.

Figure S6 shows LEED pattern of a graphene/Ir sample with submonolayer amount of Fe-TCNQ on graphene/Ir(111) compared to a sample with significant Fe-TCNQ bi-layer coverage. On both samples, there are 3 distinct orientations with respect to graphene, which are rotated by 0°, 12° and 24° from the high-symmetry axes of graphene/Ir(111) ; together with the substrate symmetry this results in 15 rotational/mirror domains observed in our experiments. On the bi-layer sample, the intensity of diffractions spots is different from the submonolayer sample, but their position appears identical.

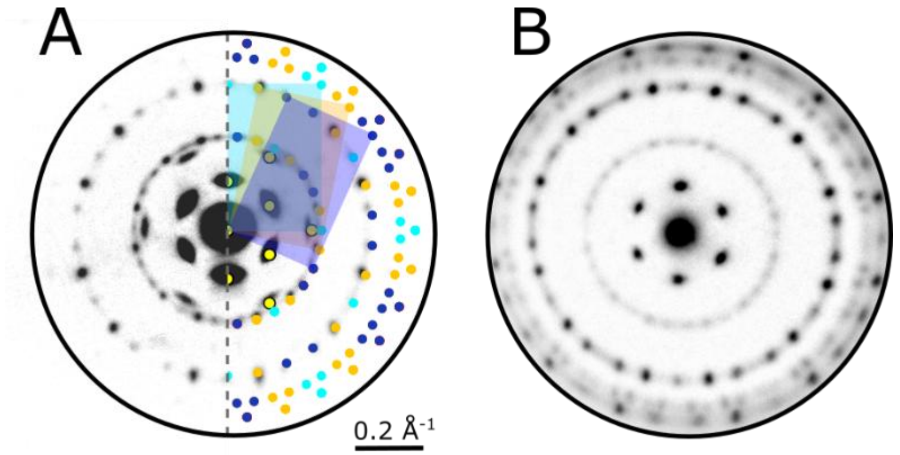

Figure S6: LEED patterns acquired on a graphene/Ir(111) sample with submonolayer amount of Fe-TCNQ (A), and >1 monolayer Fe-TCNQ with significant bi-layer coverage (B). In panel (A), the spots marked by yellow dots correspond to the graphene/Ir(111) moiré pattern, the spots marked by blue, cyan and orange spots correspond to the different rotational domains of Fe-TCNQ. The Fe-TCNQ unit cell is marked by the semi-transparent rectangles.

Supplementary Note 4:

STM appearance of the Fe-TCNQ multilayer with different tip termination and different sample biases

The out-of-plane tilt of the TCNQ linkers within multilayer Fe-TCNQ is consistently observed in STM images with varying tip termination (the exact tip termination is unknown in case of room temperature STM imaging, but the changes in contrast caused by the differing terminations are clearly observed). Figure S7 shows an STM image showing different contrast from the images shown in Figure 4 in the main text. The line profiles along the TCNQ linkers show the same appearance as the ones in Figure 4B-E, consistently indicating the out-of-plane tilt of TCNQ molecules in multilayer Fe-TCNQ.

The asymmetric appearance is not caused by the asymmetry of the tip, as proven by the comparison of the green and blue line profiles, which indicate the opposite TCNQ tilt in the two neighboring bi-layer islands. On the other hand, the tri-layer structure (pink line profile) follows the orientation of the underlying bi-layer (blue line profile).

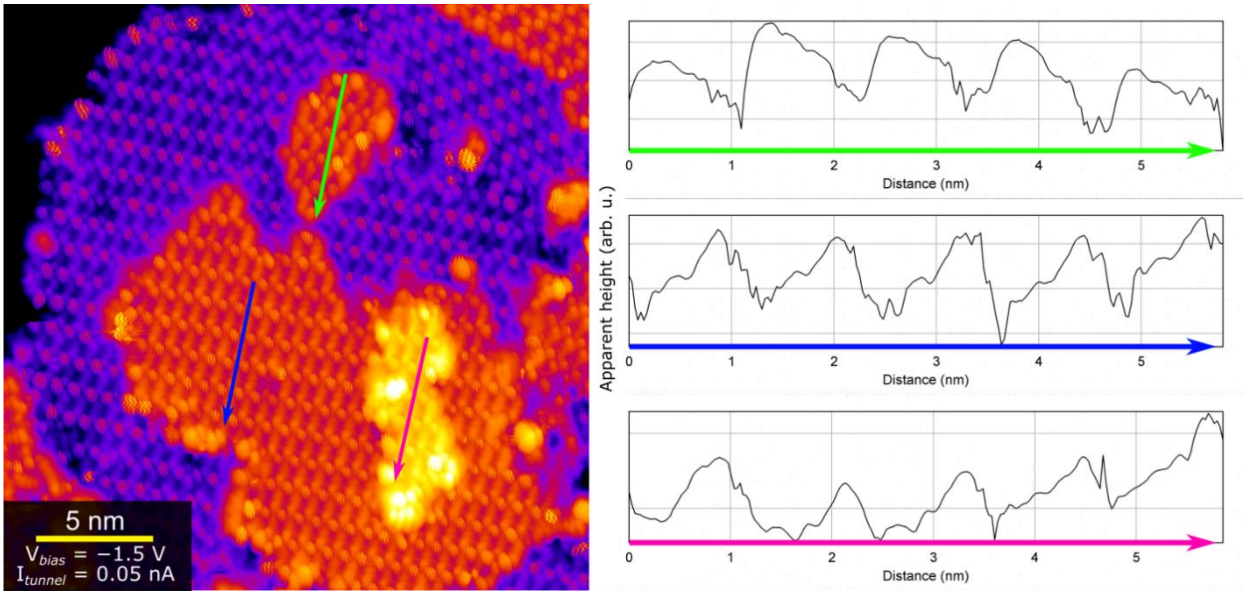

Figure S7: Apparent TCNQ tilt observed with a different tip contrast, on distinct 2nd layer islands, and on the 3rd layer.

Figure S8 shows STM images of Fe-TCNQ monolayers and bi-layers on graphene/Ir imaged with different sample biases and different tip terminations. The STM contrast is consistent with previous reports:[4] At a positive sample bias, the brightest features are the Fe cations, while the internal structure of the TCNQ molecules is poorly resolved. At negative sample biases, the signal is dominated by the states located on the TCNQ molecule. The tilt of the TCNQ molecules is best visible at sample biases around –1.3 V, where the contribution of the states located at the most protruding methyl and cyano groups is the strongest. The data

shown in Figure S8 indicate that the difference in STM contrast between monolayer and multilayer Fe-TCNQ is mostly arising from the different tilt of the TCNQ linkers. There is no major contrast difference arising from distinct electronic structure of the Fe cations or TCNQ linkers of the monolayer/multilayer areas, in agreement with the DFT analysis provided in Figure 6 in the main text.

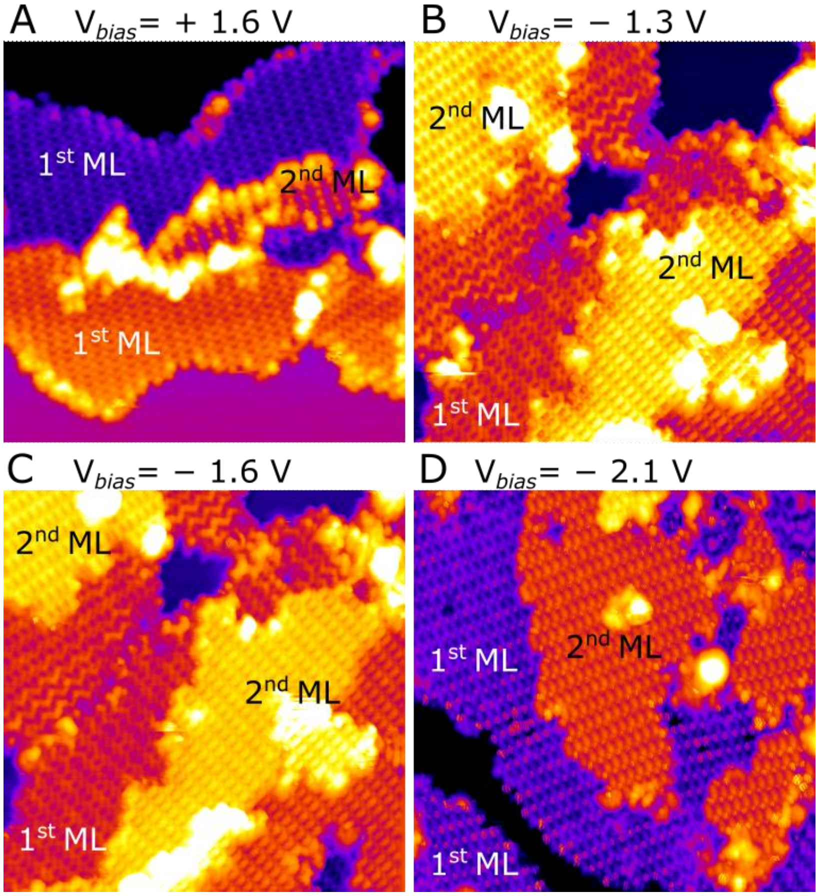

Figure S8: STM images of Fe-TCNQ monolayers and multilayers on graphene/Ir acquired at different sample biases and different tip terminations. In all panels, the scan size is 30x30 nm.

Supplementary Note 5:

DFT analysis of the inter-layer coordination bonding

To investigate the nature of the inter-layer Fe–N coordination bonding in the bilayer Fe-TCNQ phase, we performed a crystal orbital Hamilton population (COHP) analysis using the LOBSTER code.[5,6] COHP provides an energy-resolved decomposition of the electronic structure, projecting the Hamiltonian matrix elements onto specific atom–atom interactions. This allows one to distinguish bonding and antibonding contributions and to quantify the covalent character of chemical bonds.

Figure S9 shows the DFT-optimized bilayer structure together with the COHP curves for the five Fe–N bonds in the lower Fe–N_5 cluster (which is a part of the Fe-TCNQ bi-layer ground state model). The calculated bond lengths and the COHP curves for the Fe-N bonds in the upper Fe–N_5 cluster are almost identical. The bonding and antibonding contributions are highlighted in green and red, respectively, and the corresponding integrated COHP (ICOHP) values up to the Fermi level are shown for each Fe–N bond. ICOHP values of ~2.5 eV for the interlayer Fe–N bond still falls within the covalent regime, albeit slightly weaker than the intralayer Fe–N coordination bonds (2.4 – 4.0 eV). This analysis confirms that the interlayer interaction retains significant covalent character.

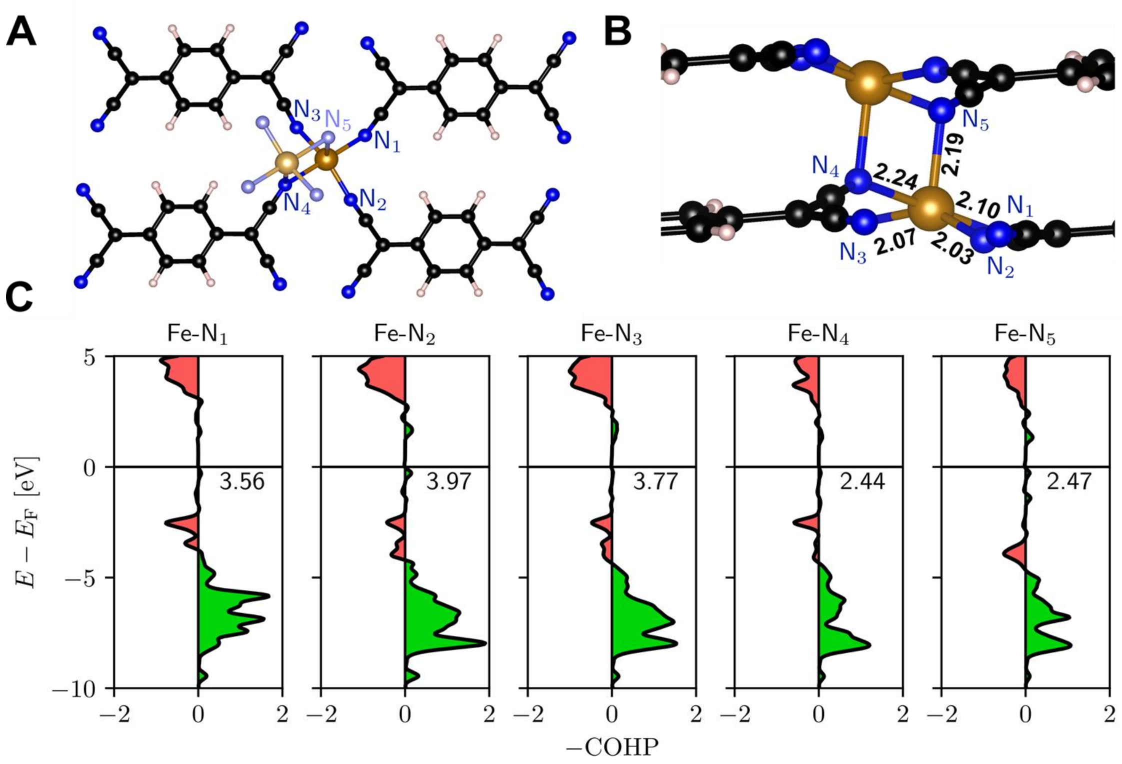

Figure S9: Inter-layer coordination bonding and calculated crystal orbital Hamilton population (COHP) curves. (A) Top view of the bottom layer of the DFT-optimized gas-phase bilayer FeTCNQ structure, with the upper Fe–N_5 cluster highlighted in lighter colors. (B) Side view of the bilayer structure, with Fe–N bond lengths for the lower Fe–N_5 cluster. (C) Calculated COHP curves for the five Fe–N bonds in the lower Fe–N_5 cluster. Bonding and antibonding contributions are shown in green and red, respectively. All panels include the corresponding integrated COHP values up to the Fermi level.

Supplementary Note 6:

Gas-phase DFT models and STM simulations

We compared different models of a Fe-TCNQ bi-layer in the gas phase, shown in Figure S10. The “tilted” and “twisted” models are based on the relaxation of the most stable Fe-TCNQ monolayer structures described in reference 4, the “5-fold” model was built by relaxing two planar Fe-TCNQ layers. Recalculating the 5-fold model by MD-MLFF simulations at 300 K leads to slight structural differences, as shown in Figure S10C,D. The STM simulations for each model are shown in the bottom half of each panel.

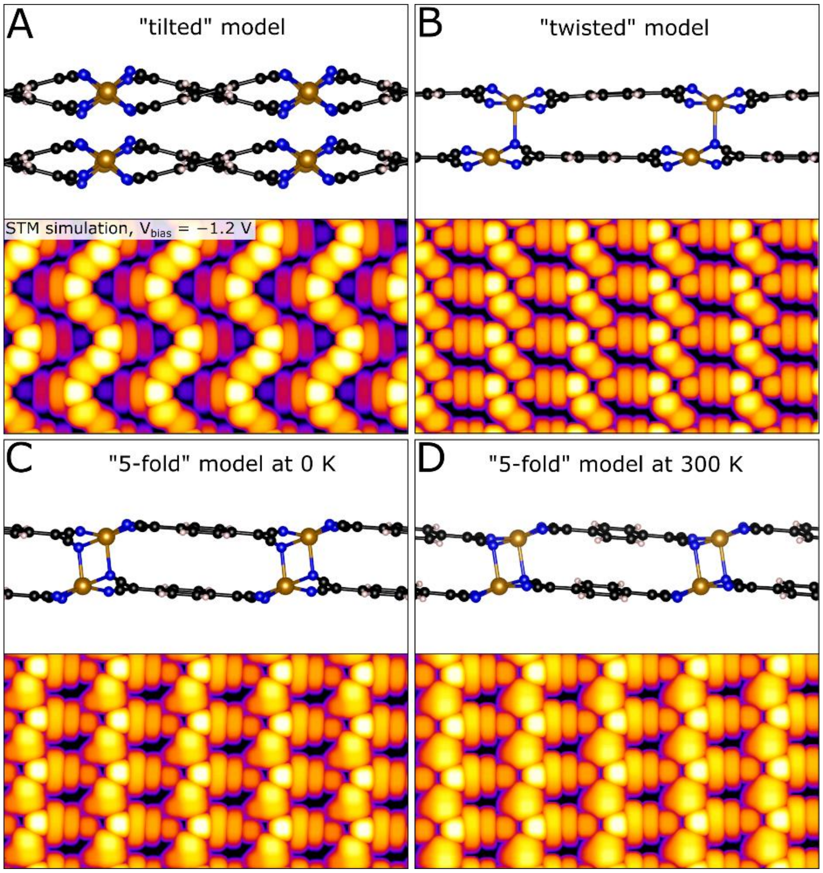

Figure S10: Gas-phase DFT-optimized models of Fe-TCNQ bilayers and the corresponding STM appearance simulated for sample bias −1.2 V.

The unit cell parameters and differences in the stabilities of the individual models are summarized in Table ST2. The “tilted” model is marginally preferred over the “5-fold”, but this is mainly allowed by the significant lateral contraction of the unit cell, which is unlikely to take place on a support. The presence of the “tilted” model in the experiment can be ruled out on the basis of experimental images shown in Figure 4 in the main text and S7, S8 in the SI. These show no resemblance of the STM simulations of the “tilted” model, but perfectly agree with the STM simulations of the “5-fold” model.

Table ST2: Stability and unit cell parameters of the gas-phase bilayer Fe-TCNQ models

Structure	E [meV/$Fe_1(TCNQ)_1$]	$a_{Fe\text{-}TCNQ}$ [Å]	$b_{Fe\text{-}TCNQ}$ [Å]	γ [˚]
Twisted	209	14.06	11.33	90.7
5-fold	27	14.44	11.31	88.7
Tilted	0	13.98	10.76	89.9

Supplementary Note 7:

Additional STM and DFT analysis of the lateral shifts between individual Fe-TCNQ layers

The lateral interlayer shift discussed in the main text (Figure 5) is consistently observed between the neighboring layers, but the magnitude and direction slightly vary in the STM images. Figure S11 shows three images of Fe-TCNQ/Gr/Ir highlighting the lateral shift. In panels A,B, the grid is aligned with the Fe cations in the 1st layer, while the orange dots highlight the positions of the Fe cations in the 2nd layer. In panel C, the grid is aligned with the Fe cations in the 2nd layer, and the yellow dots highlight the Fe cation positions in the 1st layer. The images acquired on Au(111) support show qualitatively similar stacking of Fe-TCNQ layers, but the lower achieved STM resolution does not allow to analyze the fine details of lateral shifts.

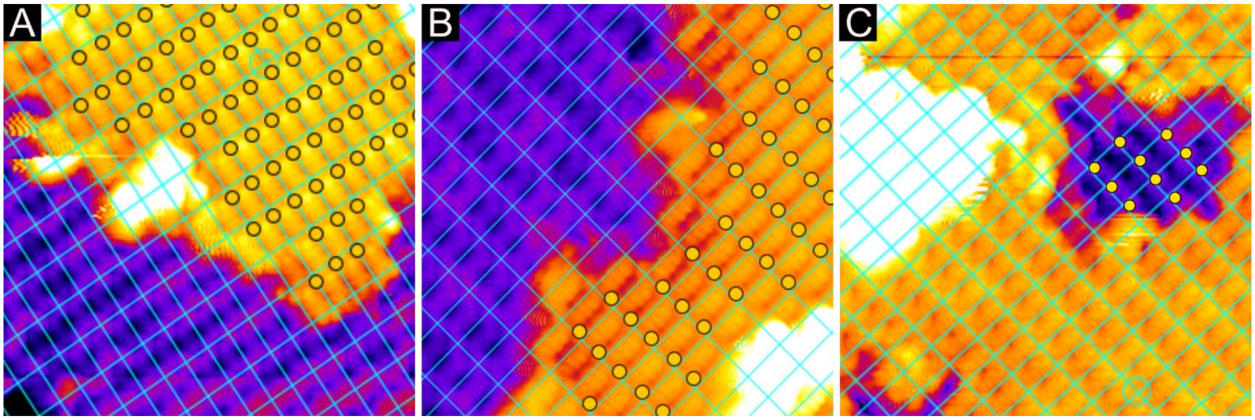

Figure S11: Interlayer shifts found analyzed on different Fe-TCNQ bilayer islands measured by STM.

We analyzed the possible interlayer shift variations by constrained DFT computations in the gas phase. Specifically, we relaxed 81 different bilayer models with fixed interlayer shifts varying in *x* and *y* directions. The energies and lateral shifts of these models are plotted in Figure S12. The black dots represent the positions of the Fe cations in the second layer with respect to Fe atoms in the first layer. The position of the underlying layer prior to relaxation is indicated by the overlaid model, and the energies of the individual laterally shifted models is

indicated by the green-red color coding (energies relative to the most stable model). The dataset shows that it is strongly unfavorable when the two Fe-TCNQ layer are aligned with the Fe cations being directly on top of each other (the red area in the center of the figure). The most stable structures feature the 2nd layer Fe atom residing above an N atom in the first layer (dark green areas at around ±1.5 Å in x, y). Nevertheless, the dataset indicates significant variability in the position around these energy minima. This may partially explain the variations observed in the experimental STM images.

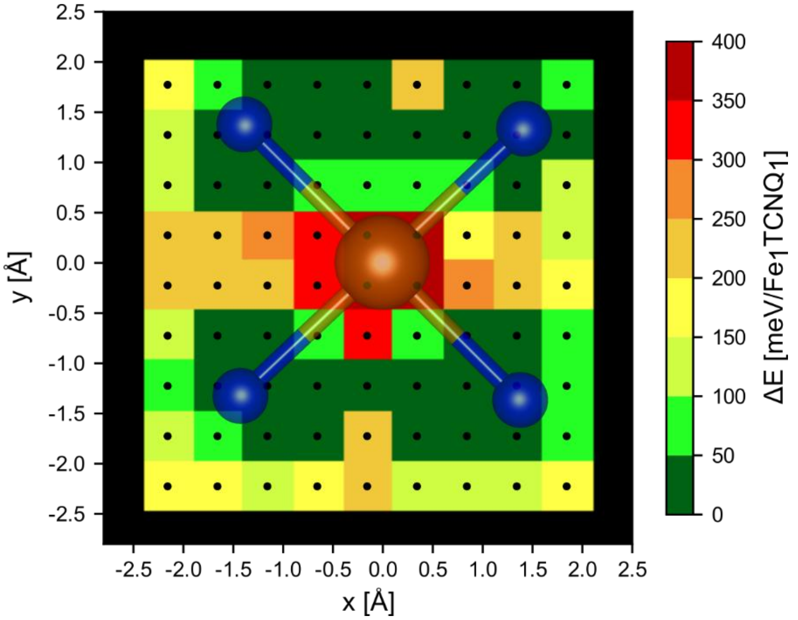

Figure S12: DFT analysis of the lateral interlayer shifts observed in the Fe-TCNQ multilayers. The black dots represent the positions of the Fe atom in the second layer relative to the Fe atom in the first layer, which are both fixed in the x and y directions during the geometry optimization. The position of the underlying layer prior to relaxation is indicated by the overlaid model, and the energies of the individual laterally shifted models is indicated by the green-red color coding.

Supplementary Note 8:

Estimation of the spring constants applied in the gas-phase MD simulations

To reduce the computational cost of the MD simulations of the graphene-supported Fe-TCNQ bilayer, the graphene support is replaced with the application of the harmonic potential acting on the z-positions of carbon atoms within the central rings of first-layer TCNQ molecules. To estimate the stiffness of the spring constant, we rotate stepwise the central rings along the long axis of the TCNQ molecule and calculate the variation of the total energy for each step (see Figure S13). This procedure is performed with and without the presence of graphene support and their differences in energy variations ΔE are assigned to the interaction of the first layer with the graphene sheet. Finally, the spring constant is

obtained from the parabolical fit of the energy difference ΔE with respect to the z-displacement of the carbon atom, i.e. $\Delta E = \frac{1}{2}k \cdot \Delta z^2$. In our case, $\Delta z = d \cdot \sin(\alpha)$, where $d = 1.21$ Å is the distance of the carbon atom from the rotational axis and α is the rotational angle. Figure S13C shows the calculated ΔE as the function of Δz with the overall stiffness constant k calculated to be 849 eV Å^{-2}. Overall, four carbon atoms are displaced by the angular rotation, and therefore, the stiffness constant of a single spring is estimated to be ~ 0.2 eVÅ^{-2}.

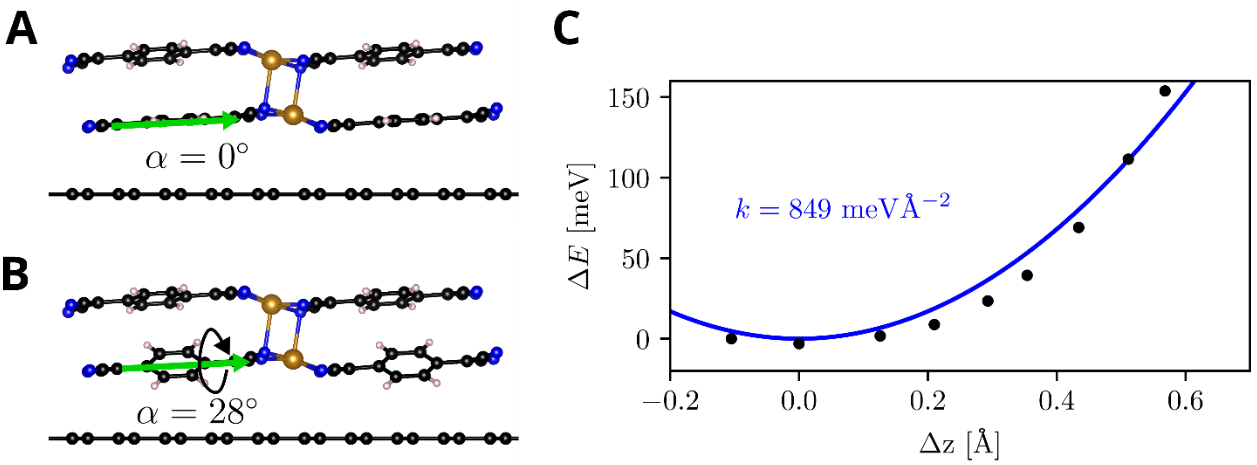

Figure S13: Estimation of the stiffness constants for the harmonic potential that simulates the presence of the graphene support. (A-B) The central rings are rotated along the axis of the TCNQ molecule (green arrows) and the differences in relative energies between the systems with and without the graphene support ΔE are plotted in panel (C) against the z-displacement of the four carbon atoms. The parabolic fit yields the stiffness constant of a single spring ~0.2eVÅ^{-2}.

Supplementary Note 9:

DFT models of bi-layer Fe-TCNQ supported on graphene

To estimate the stability of the twisted, 5-fold and tilted Fe-TCNQ phases on the incommensurate graphene support, we built five interface models with distinct Fe-TCNQ bilayer dimensions, as listed in Tables ST3 and ST4. The choice of models is such that the dimensions of the Fe-TCNQ bilayers within them are close to: (i) the gas-phase tilted Fe-TCNQ bilayer (UC 1 and UC 1B), (ii) an intermediate structure between the tilted and 5-fold Fe-TCNQ bilayers (UC 2), and (iii) the 5-fold Fe-TCNQ bilayer (UC 3 and UC 3B). All interface models and the corresponding epitaxy matrices are shown in Figure S14. Three models out of five, marked as UC 1-3, contain the Fe-TCNQ structures adjusted to match the dimensions of the graphene support. The remaining two models (UC 1B and UC 3B) contain the graphene

sheet modified to accomodate the dimensions of the gas-phase tilted Fe-TCNQ and 5-fold Fe-TCNQ bilayers, respectively.

The formation energy of each Fe-TCNQ phase in the considered unit cells is then calculated as

$$E_{form} = E_{int} - E_{gr} - E_{FeTCNQ}^{ref},$$

where E_{int} is the total energy of the Fe-TCNQ/Gr interface, E_{gr} is the reference energy of the isolated graphene sheet calculated within the same supercell and E_{FeTCNQ}^{ref} is the reference energy of the gas-phase tilted Fe-TCNQ monolayer.

The choice of the reference energy E_{gr} removes the influence of strain imposed on the graphene substrate from the calculated stability of the Fe-TCNQ bilayers. However, the impact of strain on the interaction strength between the Fe-TCNQ bilayers and the graphene sheet is still neglected. Thus, the comparison of formation energies across different unit cells should be considered as an approximate estimate.

The resulting stabilities of all Fe-TCNQ phases relative to the most stable 5-fold phase calculated in the UC 3B is given in Table ST4. The 5-fold phase is consistently more stable than the twisted bilayer across all calculated models. Furthermore, the most stable 5-fold bilayer (UC 3B) is significantly more stable (by 126 meV / $Fe_1(TCNQ)_1$) than the most stable tilted bilayer (UC 1B), suggesting the thermodynamic stability of the 5-fold bilayers. The only unit cells where the tilted phase is more stable than the 5-fold phase are the UC1 and UC1B. However, these structures are significantly laterally contracted (because they are close to the ideal dimensions of the gas-phase tilted structure), leading to strong destabilization of the 5-fold structure.

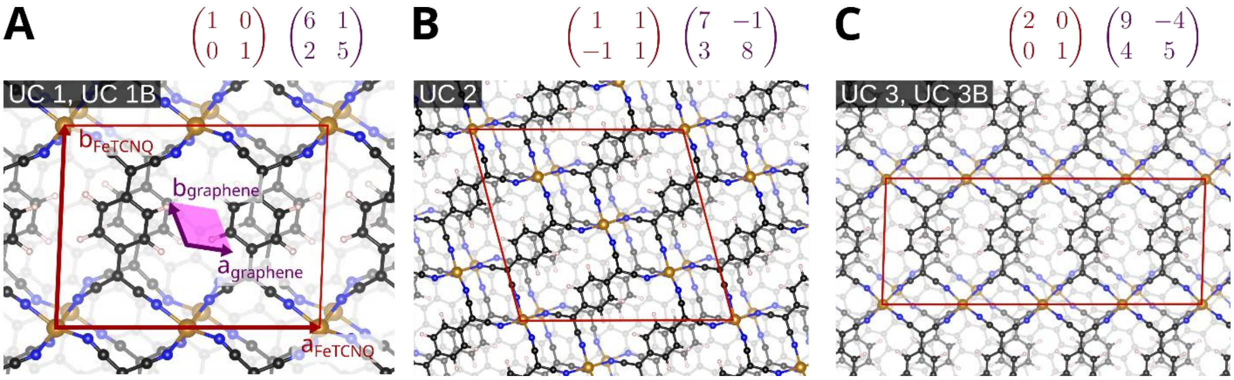

Figure S14: Calculated supercells and the corresponding epitaxy matrices for the Fe-TCNQ layer (colored with red) and for the graphene sheet (colored with violet). (A) The supercell with dimensions close to the gas-phase tilted Fe-TCNQ bilayer, marked as UC 1 and UC 1B, depending on which layer is adjusted to the supercell dimensions. (B) The supercell with dimensions close to average of the gas-phase 5-fold and tilted models. (C) The same supercell used in our previous work.[4]

Table ST3: Structural parameters of the supercells employed in DFT calculations

	a_{sup} [Å]	b_{sup} [Å]	γ_{sup} [˚]
UC 1	13.75	10.77	87.6
UC 1B	13.98	10.77	87.6
UC 2	18.65	17.29	104.8
UC 3	28.49	11.32	88.4
UC 3B	28.88	11.32	88.4

Table ST4: Structural parameters and stabilities of the individual Fe-TCNQ bilayer models computed in different supercells.

	$a_{Fe\text{-}TCNQ}$ [Å]	$b_{Fe\text{-}TCNQ}$ [Å]	γ[˚]	Strain location	Stability [meV/Fe_1TCNQ_1]		
					twisted	tilted	5-fold
UC 1	13.75	10.77	87.6	layer	370	140	266
UC 1B	13.98	10.77	87.6	substrate	336	126	215
UC 2	14.25	10.99	85.5	layer	251	163	63
UC 3	14.21	11.32	88.4	layer	80	158	10
UC 3B	14.44	11.32	88.4	substrate	38	175	0

Supplementary Note 10:

DFT analysis of the bilayer Fe-TCNQ structures on Au(111)

We evaluated the stability of several Fe-TCNQ bilayer structures on Au(111), as illustrated in Figure S15. All calculations employed the supercell and methodology described in our previous work.[4] The parameters of the unit cell (a_{sup}=18.45 Å, b_{sup}=17.72 Å, γ_{sup}=103.9˚) were fixed to those of the Au(111) substrate, resulting in $a_{Fe\text{-}TCNQ}$=14.25 Å and $b_{Fe\text{-}TCNQ}$=11.15 Å for the Fe-TCNQ layer, as shown in Figure S15A. Out of the 6 distinct bilayer configurations listed in Table ST5, the most stable structures shown in figures S15C,D exhibit intralayer coordination bonding similar to that observed for Fe-TCNQ bilayers on graphene.

Next, we evaluated the change in interaction strength (E_{int}) upon adding a second Fe-TCNQ layer on top of the monolayer, forming a Fe-TCNQ bilayer with 5-fold coordinated Fe atoms:

$$E_{int} = E_{tot} - E_{sub} - E_{FeTCNQ}$$

where E_{tot} is the total energy of the interacting system, and E_{sub} and E_{FeTCNQ} are the total energies of the isolated substrate and Fe-TCNQ layer, respectively. E_{int} decreases from 2.1 eV per Fe_1TCNQ_1 unit for the planarized monolayer structure (first Fe-TCNQ monolayer shown in Fig. S15B) to 1.52 eV for the most stable 5-fold Fe-TCNQ bilayer (Fig. S15D). This reduction arises because Fe atoms are displaced away from the Au(111) surface, forming new bonds with the second Fe-TCNQ layer. This weakening of the Fe-TCNQ/Au(111) interaction may be one of the reasons for the experimental observation of different number of rotational domains of monolayer and multilayer Fe-TCNQ on Au(111).

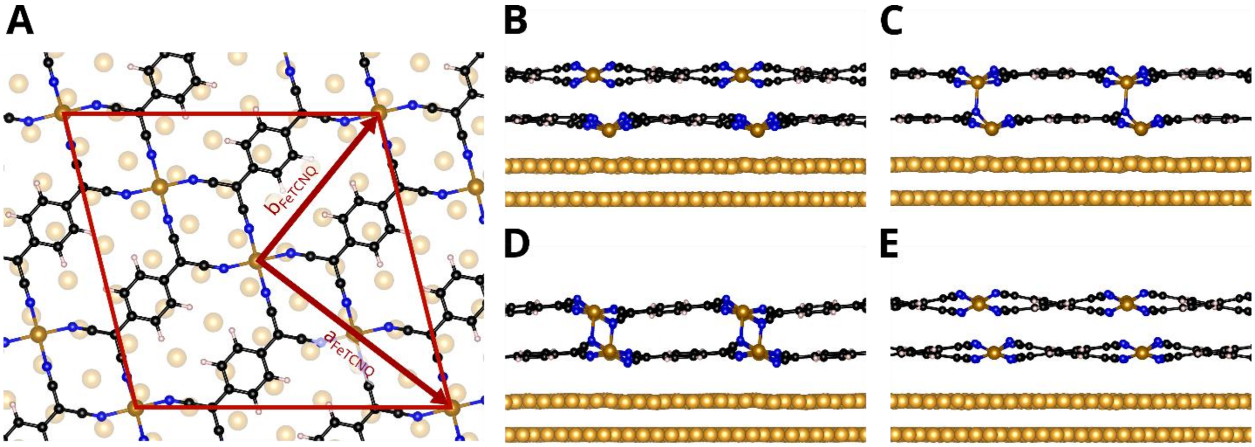

Figure S15: DFT models of Fe-TCNQ multilayers on Au(111). (A) Top view of a planarized Fe-TCNQ monolayer on Au(111). Red arrows indicate the unit cell vectors of the Fe-TCNQ layer. (B–E) Calculated bilayer models: (B) tilted Fe-TCNQ monolayer on a planarized monolayer; (C) 5-fold Fe-TCNQ monolayer on a planarized Fe-TCNQ monolayer; (D) 5-fold Fe-TCNQ bilayer; (E) tilted Fe-TCNQ bilayer.

Table ST5: Relative stabilities of the Fe-TCNQ/Au supercells employed in DFT calculations.

Structure	Stability [meV/Fe_1TCNQ_1 unit]
tilted + planarized Fe-TCNQ [Fig. S15B]	51
5-fold + planarized Fe-TCNQ [Fig. S15C]	17
5-fold Fe-TCNQ bilayer [Fig. S15D]	0
tilted Fe-TCNQ bilayer [Fig. S15E]	78
twisted Fe-TCNQ bilayer	unstable
planar Fe-TCNQ bilayer	unstable

Supplementary Note 11:

Electronic properties of the Fe-TCNQ structures supported on graphene

Table ST6 summarizes the calculated d-band occupancy, magnetization, and Bader charges of Fe atoms in the Fe-TCNQ models supported on graphene shown in Figure 6 in the main text. The results indicate that the Fe atoms retain a high spin d^6 configuration in all the tested models, as coordination geometry is changed from 4-fold quasi-tetrahedral in the tilted structures to 5-fold distorted square-pyramidal and 6-fold octahedral in the multilayer structures.

Table ST6: Calculated occupancy, magnetization and Bader charges of Fe atoms for DFT models shown in Figure S6.

Structure (geometry) of Fe	majority spin Fe d electrons	minority spin Fe d electrons	Total occupied Fe d electrons	Bader charge
4-fold Fe in monolayer	4.85	1.20	6.05	1.37
5-fold Fe in bi-layer	4.85	1.21	6.06	1.37
4-fold Fe in hypothetical bi-layer without interlayer bonding	4.84	1.19	6.03	1.39
5-fold Fe in tri-layer	4.85	1.21	6.06	1.39
6-fold Fe in tri-layer	4.87	1.19	6.06	1.40

References

(1) Schmid, M.; Zirzlmeier, J.; Steinrück, H.-P.; Gottfried, J. M. Interfacial Interactions of Iron(II) Tetrapyrrole Complexes on Au(111). *J. Phys. Chem. C* **2011**, *115* (34), 17028–17035.

(2) Grosvenor, A. P.; Kobe, B. A.; Biesinger, M. C.; McIntyre, N. S. Investigation of Multiplet Splitting of Fe 2p XPS Spectra and Bonding in Iron Compounds. *Surf. Interface Anal.* **2004**, *36* (12), 1564–1574.

(3) Blowey, P. J.; Velari, S.; Rochford, L. A.; Duncan, D. A.; Warr, D. A.; Lee, T. L.; De Vita, A.; Costantini, G.; Woodruff, D. P. Re-Evaluating How Charge Transfer Modifies the Conformation of Adsorbed Molecules. *Nanoscale* **2018**, *10* (31), 14984–14992.

(4) Jakub, Z.; Shahsavar, A.; Planer, J.; Hrůza, D.; Herich, O.; Procházka, P.; Čechal, J. How the Support Defines Properties of 2D Metal–Organic Frameworks: Fe-TCNQ on Graphene versus Au(111). *J. Am. Chem. Soc.* **2024**, *146* (5), 3471–3482.

(5) Dronskowski, R.; Bloechl, P. E. Crystal Orbital Hamilton Populations (COHP): Energy-Resolved Visualization of Chemical Bonding in Solids Based on Density-Functional Calculations. *J. Phys. Chem.* **1993**, *97* (33), 8617–8624.

(6) Maintz, S.; Deringer, V. L.; Tchougréeff, A. L.; Dronskowski, R. LOBSTER: A Tool to Extract Chemical Bonding from Plane-Wave Based DFT. *J. Comput. Chem.* **2016**, *37* (11), 1030–1035.
